# The gene Sr38 for bread wheat breeding in Western Siberia

**DOI:** 10.18699/VJ21.084

**Published:** 2021-11

**Authors:** E.S. Skolotneva, V.N. Kelbin, V.P. Shamanin, N.I. Boyko, V.A. Aparina, E.A. Salina

**Affiliations:** Institute of Cytology and Genetics of the Siberian Branch of the Russian Academy of Sciences, Novosibirsk, Russia; Institute of Cytology and Genetics of the Siberian Branch of the Russian Academy of Sciences, Novosibirsk, Russia; Omsk State Agrarian University named after P.A. Stolypin, Omsk, Russia; Siberian Research Institute of Plant Production and Breeding – Branch of the Institute of Cytology and Genetics of the Siberian Branch of the Russian Academy of Sciences, Novosibirsk, Russia; Siberian Research Institute of Plant Production and Breeding – Branch of the Institute of Cytology and Genetics of the Siberian Branch of the Russian Academy of Sciences, Novosibirsk, Russia; Institute of Cytology and Genetics of the Siberian Branch of the Russian Academy of Sciences, Novosibirsk, Russia; Kurchatov Genomic Center of ICG SB RAS, Novosibirsk, Russia

**Keywords:** Puccinia graminis f. sp. tritici, avirulent clones, resistance, Triticum aestivum, Sr38, Puccinia graminis f. sp. tritici, авирулентные клоны, устойчивость, Triticum aestivum, Sr38

## Abstract

Present-day wheat breeding for immunity exploits extensively closely related species from the family Triticeae as gene donors. The 2NS/2AS translocation has been introduced into the genome of the cultivated cereal Triticum aestivum from the wild relative T. ventricosum. It contains the Lr37, Yr17, and Sr38 genes, which support seedling resistance to the pathogens Puccinia triticina Eriks., P. striiformis West. f. sp. tritici, and P. graminis Pers. f. sp. tritici Eriks. & E. Henn, which cause brown, yellow, and stem rust of wheat, respectively. This translocation is present in the varieties Trident, Madsen, and Rendezvous grown worldwide and in the Russian varieties Morozko, Svarog, Graf, Marquis, and Homer bred in southern regions. However, the Sr38 gene has not yet been introduced into commercial varieties in West Siberia; thus, it remains of practical importance for breeding in areas where populations of P. graminis f. sp. tritici are represented by avirulent clones. The main goal of this work was to analyze the frequency of clones (a)virulent to the Sr38 gene in an extended West Siberian collection of stem rust agent isolates. In 2019–2020, 139 single pustule isolates of P. graminis f. sp. tritici were obtained on seedlings of the standard susceptible cultivar Khakasskaya in an environmentally controlled laboratory (Institute of Cytology and Genetics SB RAS) from samples of urediniospores collected on commercial and experimental bread wheat f ields in the Novosibirsk, Omsk, Altai, and Krasnoyarsk regions. By inoculating test wheat genotypes carrying Sr38 (VPM1 and Trident), variations in the purity of (a)virulent clones were detected in geographical samples of P. graminis f. sp. tritici. In general, clones avirulent to Sr38 constitute 60 % of the West Siberian fungus population, whereas not a single virulent isolate was detected in the Krasnoyarsk collection. The Russian breeding material was screened for sources of the stem rust resistance gene by using molecular markers specif ic to the 2NS/2AS translocation. A collection of hybrid lines and varieties of bread spring wheat adapted to West Siberia (Omsk SAU) was analyzed to identify accessions promising for the region. The presence of the gene was postulated by genotyping with specif ic primers (VENTRIUP-LN2) and phytopathological tests with avirulent clones of the fungus. Dominant Sr38 alleles were identif ied in Lutescens 12-18, Lutescens 81-17, Lutescens 66-16, Erythrospermum 79/07, 9-31, and 8-26. On the grounds of the composition of the West Siberian P. graminis f. sp. tritici population, the Sr38 gene can be considered a candidate for pyramiding genotypes promising for the Novosibirsk, Altai, and Krasnoyarsk regions

## Abstract (ru)

Современная селекция пшеницы на иммунитет широко применяет генетический резерв близкородственных видов из семейства Triticeae. Транслокация 2NS/2AS привнесена в геном культурного злака Triticum aestivum от дикорастущего сородича T. ventricosum и содержит гены Lr37, Yr17 и Sr38, которые отвечают за устойчивость пшеницы на уровне проростков к бурой, желтой и стеблевой ржавчине с соответствующими возбудителями: Puccinia triticina Eriks., P. striiformis West. f. sp. tritici и P. graminis Pers. f. sp. tritici Eriks. & E. Henn. Данная транслокация известна в таких мировых сортах, как Trident, Madsen, Rendezvous, а также в отечественных сортах южной селекции Морозко, Сварог, Граф, Маркиз и Гомер. При этом ген Sr38 до сих пор не введен в производственные сорта, высеваемые на территории Западной Сибири, поэтому сохраняет практическое значение для селекции на иммунитет в областях, где патогенная популяция P. graminis f. sp. tritici представлена авирулентными клонами. Основная цель работы состояла в анализе частоты а/вирулентных клонов к гену Sr38 в расширенной западносибирской выборке возбудителя стеблевой ржавчины. В лаборатории с контролируемым климатом (Институт цитологии и генетики СО РАН) на проростках универсального восприимчивого сорта Хакасская выделено 139 монопустульных изолятов P. graminis f. sp. tritici из образцов урединиоспор Новосибирской, Омской областей, Алтайского и Красноярского края, собранных в 2019–2020 гг. на производственных и селекционных посевах мягкой пшеницы. Путем заражения тестерных генотипов пшеницы, несущих ген Sr38 (VPM1 и Trident), выявлены вариации по частоте а/вирулентных клонов в географических образцах P. graminis f. sp. tritici. В целом текущая западносибирская популяция представлена на 60 % авирулентными клонами гриба к гену Sr38, при этом в образцах популяции из Красноярского края не выявлено ни одного вирулентного изолята. Поиск источников гена устойчивости к стеблевой ржавчине среди отечественного селекционного материала был выполнен с помощью специфических молекулярных маркеров на транслокацию 2NS/2AS. Исходя из перспективы использования в регионе, выбор проводили среди коллекции линий и сортов мягкой яровой пшеницы Омского ГАУ, адаптированных к условиям Западной Сибири. Присутствие гена постулировалось путем проведения процедуры генотипирования с помощью специфических праймеров (VENTRIUP-LN2) и фитопатологического тестирования авирулентными клонами гриба. Носителями доминантных аллелей гена Sr38 оказались линии Лютесценс 12-18, Лютесценс 81-17, Лютесценс 66-16, Эритроспермум 79/07, 9-31 и 8-26. Полученные данные по составу образцов западносибирской популяции P. graminis f. sp. tritici позволяют рассматривать ген Sr38 в качестве кандидата для включения в селекцию пшеницы в Красноярском крае, а также в составе генных пирамид в Новосибирской области и Алтайском крае.

## Introduction

countries for millennia. The exhaustion of the diversity of wheat genes potentially encoding commercially valuable traits, including pest resistance, is inevitable. Wild relatives in the Triticeae family are broadly used as genetic resources for modern wheat breeding for immunity. They include Triticum monococcum L., T. speltoides (Tausch) Gren., and T. ventricosum (McIntosh et al., 1995; Dubcovsky et al., 1996; Friebe et al., 1996). A long chromosome stretch (25–38 cM) hosting three genes for rust resistance was transferred to the genome of bread wheat variety VPM1 from T. ventricosum (Maia, 1967) and identified as a 2NS/2AS translocation (Bariana, McIntosh, 1993). The acquired genes Lr37, Yr17, and Sr38 confer resistance against brown, yellow, and stem rusts, caused by Puccinia triticina Eriks., P. striiformis West. f. sp. tritici, and P. graminis Pers. f. sp. tritici Eriks. & E. Henn., respectively. The 2NS/2AS translocation was also introgressed to other commercial varieties: Trident, Madsen, and Rendezvous (McIntosh et al., 1995). Then it was extensively used in breeding in various regions of the world, where it provided efficient protection from rust agents and some nematode species attacking cereals (Dyck, Lukow, 1988; Robert et al., 1999; Seah et al., 2000). Cultivars with the identified Lr37 gene for brown rust resistance and, correspondingly, with the Sr38 and Yr17 genes for resistance to stem and yellow rusts were raised at the Lukyanenko National Center of Grain, put on the Russian state register, and authorized for commercial use in the Central Chernozem, North Caucasian, Middle Volga, and Lower Volga regions. They include Morozko (2015), Svarog (2017), Graf (2018), Marquis (2019), and Homer (2020) (Bespalova et al., 2019a, b).

The Sr38 gene became inefficient against stem rust in countries of Asia and Northern Africa when the aggressive southern race Ug99 started its expansion (Pretorius et al., 2000). However, this race has not yet been detected among wheat pathogens in Russia (Baranova et al., 2015; Skolotneva et al., 2020b). Moreover, it has been shown that low temperatures enhance Sr38 expression (Helguera et al., 2003). Thus, it may be promising in wheat breeding in regions with temperate climate. As Sr38 has not been widely introduced into commercial varieties grown in West Siberia (Sochalova, Lichenko, 2015), is remains of practical significance for breeding for resistance in regions where pathogenic P. graminis f. sp. tritici populations are represented by avirulent clones.

Several molecular markers of the 2NS/2AS translocation have been designed to facilitate the transfer of the Lr37, Yr17, and Sr38 genes to commercial varieties. The first of the proposed markers was the dominant SCAR (Sequence Characterized Amplified Region) marker, located at 0.8 ± 0.7 cM apart from the Yr17 gene (Robert et al., 1999). At present, two markers are widely used to identify the 2NS/2AS translocation in wheat genetic material (Helguera et al., 2003). The codominant CAPS (Cleavage Amplified Polymorphic Sequence) marker demands an additional step of digesting the diagnostic fragment with restriction endonucleases. The dominant PCR marker is targeted directly at a specific sequence of the typical allele inside the translocation. The amplification is done with the VENTRIUP-LN2 primer pair, and the products are resolved in agarose gel (https://maswheat.ucdavis.edu/ protocols/Sr38), which is an obvious advantage of the marker.

Here we analyze the frequencies of clones (a)virulent against Sr38 in a West Siberian collection of stem rust agent isolates extended by adding samples from the Krasnoyarsk region. Another objective of this work is the DNA markerassisted search for Sr38-carrying accessions. The study involved a collection of spring bread wheat lines and cultivars adapted for growing in West Siberia.

## Materials and methods

The extended West Siberian collection of the stem rust agent included samples from the Novosibirsk, Omsk, Altai, and Krasnoyarsk regions collected from commercial and experimental bread wheat fields in 2019–2020. A total of 139 P. graminis f. sp. tritici single pustule isolates were obtained from the collected urediniospores on seedlings of the standard susceptible cultivar Khakasskaya in an environmentally controlled laboratory (Institute of Cytology and Genetics, Novosibirsk) (Table 1).

**Table 1.**
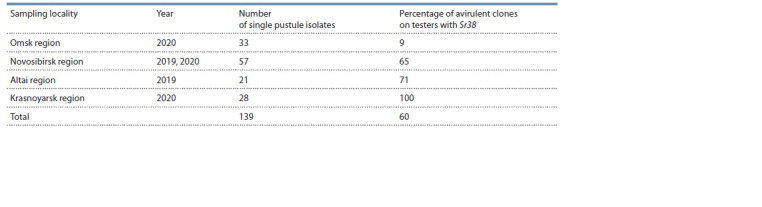
Percentages of avirulent P. graminis f. sp. tritici clones on Sr38-bearing tester wheat varieties

The frequencies of clones avirulent to the Sr38 gene were determined on tester wheat genotypes: an isogenic line and varieties from a set for differentiating stem rust races on wheats of the USA and Canada bearing the Sr38 gene: VPM1 and Trident, respectively. Prior to the experiment, the seed material was verified with molecular markers to the gene, and plants Sr38-negative on the DNA array were rejected.

The protocols for seedling preparation and inoculation with fungus clones for the analysis of resistance are described in detail by Skolotneva et al. (2020a). The infection types on wheat tester lines were scored according to the Stackman four-point scale (Stackman et al., 1962).

The collection of 80 bread wheat lines and varieties adapted to the West Siberian conditions was kindly provided by Prof. V.P. Shamanin, Omsk SAU. DNA was isolated from seedling apices by the CTAB method (Rogers, Bendich, 1985). DNA was quantified with a Qubit 4 fluorometer (Invitrogen, United States).

The Sr38 gene was identified in the material with the primers VENTRIUP (5′-AGGGCTACTGACCAAGGCT-3′) and LN2 (5′-TGCAGCTACAGCAGTATGTACACAAAA-3′) for the 2NS/2AS translocation. Amplification mixture: 1× SE- buffer AS (ammonium sulfate), 0.2 mM each dNTP, 0.2 μM each primer, 1.5 mM MgCl2, 50 ng of genomic DNA, 1 U of Taq DNA polymerase (SibEnzyme, Russia), volume 25 μL. The reaction was carried out in a Bio-Rad T100 thermocycler (United States) according to the following program: predenaturation 7 min at 94 °C followed by 30 cycles: 94 °C, 30 s; 65 °C, 30 s; 72 °C, 30 s. Postextension was performed at 72 °C for 10 min. The products were resolved in 2 % agarose gel. Fragment sizes were assessed against the Step 50 plus DNA ladder (Biolabmix, Russia).

The final step of gene postulation was the phytopathological test of resistance with P. graminis f. sp. tritici isolates avirulent against Sr38. Plant resistance was assessed at the seedling stage as mentioned above. The Khakasskaya variety was chosen as the susceptible control. The experiment was carried out on ten plants of each genotype in two replications.

## Results and discussion

**Fig. 1.**
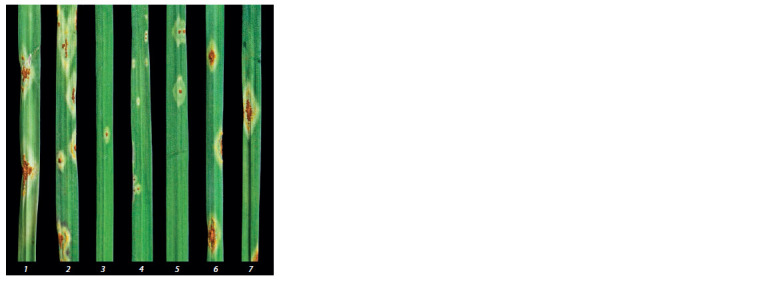
Infection types of P. graminis f. sp. tritici from various regions tested on genotypes with Sr38. Reaction type scores with fungus isolates: from the Novosibirsk region: (1) 3+, (2) 3–, (3) 1; from the Altai region: (4) 1; from the Krasnoyarsk region: (5) 2; from the Omsk region: (6) 3, (7) 3+.

While assessing stem rust agent isolates from various localities in West Siberia, we detected a variation in the frequencies of fungus clones not attacking tester genotypes with Sr38, that is, avirulent against them (see Table 1). The variation showed a longitudinal cline from the minimum frequency in the Omsk region to the nearly 100 % avirulence in the population of the Krasnoyarsk region. The polymorphism of the detected infection types in response to the inoculation with single pustule P. graminis f. sp. tritici isolates from different samples is illustrated in Figure 1. All types scoring 1, 2, 3, and 3+ were detected, but those corresponding to resistance and medium resistance were predominant in isolates from the Altai and Krasnoyarsk regions. Noteworthy is the occurrence of avirulent clones in the Novosibirsk and Altai samples, not observed in the analysis of the races of the West Siberian population in 2017 (Skolotneva et al., 2020b). This fact may be due to importation of P. graminis f. sp. tritici inoculum from southern regions. It is known that the Sr38 gene is efficient in northern Kazakhstan and China (Koyshybaev, 2018; Li et al., 2018)

In general, clones avirulent against Sr38 constitute 60 % of the West Siberian population. If we reject the collection from the Omsk region, where the gene has been considered inefficient against the local agent for several years (Shamanin et al., 2020), the frequency of fungus clones not injuring genotypes with Sr38 increases to 78 %. Therefore, the gene can be useful in gene pyramiding for eastern West Siberia. The efficiency of the genotypes Sr25+Sr38 and Sr31+Sr38 has been demonstrated in the Urals, where Sr38 alone cannot sufficiently protect plants from stem rust (Druzhin et al., 2018). An additional valuable feature of the 2NS/2AS translocation is that it bears the resistance genes Lr37 and Yr17, which remain efficient against West Siberian isolates of brown and yellow rust agents (Skolotneva et al., 2018; Gultyaeva, Shaydayuk, 2020).

Donors of the Sr38 gene were sought in the Russian breeding material with a specific molecular marker for the 2NS/2AS translocation. As the breeding programs should be targeted at West Siberia, the Omsk SAU collection of spring bread wheat lines and varieties adapted to the region was screened. The gene presence was postulated by genotyping with specific primers (VENTRIUP-LN2) and phytopathological tests with avirulent fungus clones.

**Fig. 2.**
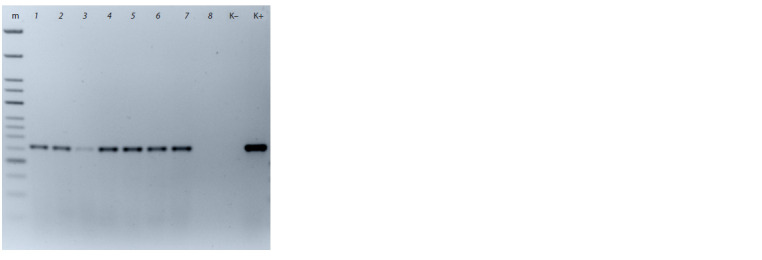
Electrophoretic image of amplification with molecular markers to the Sr38 gene on bread wheat DNA from the West Siberian collection of experimental lines, Omsk SAU. Lanes: m, Step 50 plus DNA ladder (Biolabmix); 1, Lutescens 12-18; 2, Lutescens 34-16; 3, Lutescens 81-17; 4, Lutescens 66-16; 5, Erythrospermum 79/07; 6, line 9-31; 7, line 8-26; 8, genotype 2 from the Omsk SAU collection; “C–”, negative control (cv. Khakasskaya); “C+”, positive control (VPM1).

Positive signals corresponding to the diagnostic 259 bp long amplicon were obtained from DNA templates of seven experimental wheat lines: Lutescens 12-18, Lutescens 34-16, Lutescens 81-17, Lutescens 66-16, Erythrospermum 79/07, 9-31, and 8-26 (Fig. 2). The pedigrees of these varieties and hybrid lines are shown in Table 2. The dramatic variation in the origins of the supposed Sr38 carriers deserves special attention, as it augments the value of the accessions as diverse resistance donors.

**Table 2.**
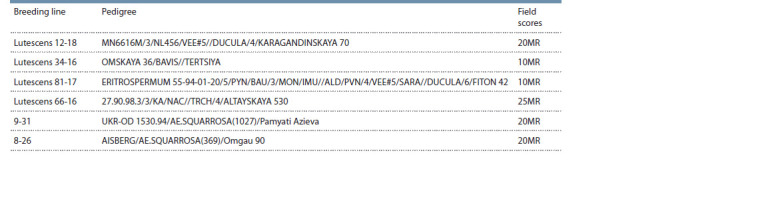
Pedigrees of some wheat lines from the West Siberian collection (Omsk SAU) resistant to stem rust against the natural infectious background of the Omsk region, 2019

**Fig. 3.**
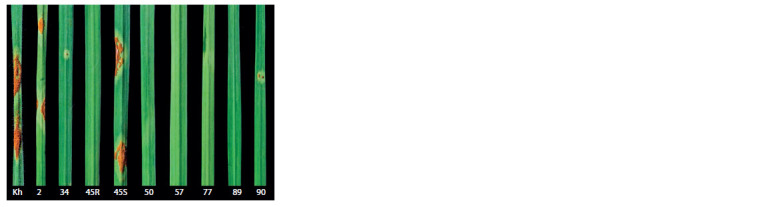
Reaction type scores of bread wheat cultivars and hybrids from the Omsk SAU West Siberian collection inoculated with P. graminis f. sp. tritici isolates avirulent against Sr38. Seedlings: Kh, cv. Khakasskaya (score 4); 2, genotype 2 from the Omsk SAU collection (score 3); 34, Lutescens 12-18 (score 1); 45R and 45S, Lutescens 34- 16 (scores 0 and 4, respectively); 50, Lutescens 81-17 (score 0); 57, Lutescens 66- 16 (score 0), 77, Erythrospermum 79/07 (score 0); 89, line 9-31 (score 0); 90, line 8-26 (score 1).

Puccinia graminis f. sp. tritici isolates eliciting stable responses on Sr38-bearing tester wheat genotypes were picked from infection samples of the Krasnoyarsk region for phytopathological tests of the West Siberian collection of bread wheat cultivars and hybrids. Infection types 0 and 1, indicative of resistance, were observed on inoculated plants of Lutescens 12-18, Lutescens 34-16, Lutescens 81-17, Lutescens 66-16, Erythrospermum 79-07, 9-31, and 8-26 (Fig. 3). In addition to the susceptible control (cv. Khakasskaya), we added for reference genotype 2, which lacks Sr38 according to genotyping with molecular markers. They showed the maximum development of stem rust signs, scored 3 and 4. Part of the tested Lutescens 34-16 plants were susceptible to fungal isolates avirulent against Sr38 (45S and 45R in Fig. 3). They constituted 30 % of the tested sample. This observation indicates that the breeding material contained biotypes differing in stem rust resistance. The molecular marker is dominant; therefore, it cannot rule out heterozygosity for the character, as found in phytopathological tests. The presence of resistant Sr38 alleles, expressing in response to the infection by avirulent clones of the fungus in accordance with Flor’s gene-for-gene relationship, describing the interaction between a host and a pathogen, was proven in the remaining West Siberian bread wheat accessions: Lutescens 12-18, Lutescens 81-17, Lutescens 66-16, Erythrospermum 79/07, 9-31, and 8-26. The results of immunological screening of these lines in field tests of breeding material against the natural infectious background point to medium stem rust resistance in Sr38 carriers (see Table 2). This fact is consistent with phytopathological tests on seedlings with isolates from the Omsk P. graminis f. sp. tritici population. 

## Conclusion

The analysis of West Siberian P. graminis f. sp. tritici isolates shows that the Sr38 gene is promising for wheat breeding in the Krasnoyarsk region and for gene pyramiding in the Novosibirsk and Altai regions. The following bread wheat cultivars and experimental lines from the Omsk SAU collection carry dominant Sr38 alleles: Lutescens 12-18, Lutescens 81-17, Lutescens 66-16, Erythrospermum 79/07, 9-31, and 8-26. These accessions are adapted to the regional environment; therefore, they may be recommended as stem rust resistance donors for breeding programs in West Siberia.

## Conflict of interest

The authors declare no conflict of interest.
